# Harnessing RNA interference for the control of *Fusarium* species: A critical review

**DOI:** 10.1111/mpp.70011

**Published:** 2024-10-04

**Authors:** Caihong Liu, Karl‐Heinz Kogel, Maria Ladera‐Carmona

**Affiliations:** ^1^ Institute of Phytopathology, Research Centre for BioSystems, Land Use and Nutrition Justus Liebig University Giessen Giessen Germany; ^2^ Institut de Biologie Moléculaire des Plantes, CNRS Université de Strasbourg Strasbourg France

**Keywords:** *Fusarium*, host‐induced gene silencing (HIGS), microbe‐induced gene silencing (MIGS), post‐transcriptional gene silencing, RNA interference (RNAi), spray‐induced gene silencing (SIGS), virus‐induced gene‐silencing system (VIGS)

## Abstract

*Fusarium* fungi are a pervasive threat to global agricultural productivity. They cause a spectrum of plant diseases that result in significant yield losses and threaten food safety by producing mycotoxins that are harmful to human and animal health. In recent years, the exploitation of the RNA interference (RNAi) mechanism has emerged as a promising avenue for the control of *Fusarium*‐induced diseases, providing both a mechanistic understanding of *Fusarium* gene function and a potential strategy for environmentally sustainable disease management. However, despite significant progress in elucidating the presence and function of the RNAi pathway in different *Fusarium* species, a comprehensive understanding of its individual protein components and underlying silencing mechanisms remains elusive. Accordingly, while a considerable number of RNAi‐based approaches to *Fusarium* control have been developed and many reports of RNAi applications in *Fusarium* control under laboratory conditions have been published, the applicability of this knowledge in agronomic settings remains an open question, and few convincing data on RNAi‐based disease control under field conditions have been published. This review aims to consolidate the current knowledge on the role of RNAi in *Fusarium* disease control by evaluating current research and highlighting important avenues for future investigation.

## Graphical abstract

We review biotechnology‐based crop protection against Fusarium diseases with novel RNA delivery technologies and RNA active ingredients.

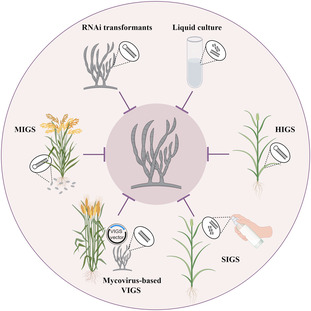

## 1 INTRODUCTION

*Fusarium* species represent one of the most important groups of plant‐pathogenic fungi that profoundly impact agricultural production across the globe. The genus *Fusarium* has a wide variety of species, comprising at least 300 phylogenetically distinct species along with 20 species complexes and nine monotypic lineages (O'Donnell et al., 2022). A great number of them cause devastating diseases in a wide range of agricultural and horticultural crops. An assessment of the American Phytopathology Society website revealed that 83 out of 108 plant species suffer from one or more *Fusarium* diseases, impacting their production (Summerell, 2019). *Fusarium oxysporum*, one of the top 10 most important pathogens, even possesses the capability to infect over 100 different host plants, causing some of the most important plant diseases including Panama disease, Fusarium wilt of tomato and soybean, as well as root rot disease of rice (Dean et al., 2012; Michielse & Rep, 2009). Another species included in the top ten rankings is *Fusarium graminearum*, which is highly destructive to cereal species, predominantly causing the Fusarium head blight (FHB) and Fusarium seedling blight (FSB), particularly prevalent in regions with temperate climates (Chen et al., 2019; Savary et al., 2012), while *F. pseudograminearum* causes Fusarium crown rot (FCR) and Fusarium root rot (FRR) in cereals and is particularly problematic in regions with semi‐arid or Mediterranean climates (Alahmad et al., 2018). Other *Fusarium* species such as *Fusarium culmorum*, *Fusarium verticillioides*, *Fusarium asiaticum* and *Fusarium solani* also cause FHB or Fusarium wilt, leading to substantial economic losses worldwide (Laraba et al., 2017; Mu et al., 2019; Shanmugam et al., 2017; Xu et al., 2021). However, the threat posed by *Fusarium* species does not stop here—some of them produce a cocktail of mycotoxins. In the latest BIOMIN World Mycotoxin Survey (https://www.biomin.net/en/biomin‐mycotoxin‐survey/), deoxynivalenol (DON) was one of the most abundant mycotoxins in the agricultural commodities used for livestock feed from 78 countries worldwide. The occurrence of mycotoxins in processed feeds and foods poses a significant threat to human consumption, animal feed and malting purposes (O'Donnell et al., 2018; van Egmond et al., 2007).

So far, control of *Fusarium* infections remains problematic. Except for changes to cultural practices and crop rotation, crop breeding is one of the main methods for disease management. However, challenges such as the extensive range of hosts of *Fusarium* species (Ma et al., 2013; Summerell, 2019), the presence of new virulent pathogenic races (Epstein et al., 2022; McGovern, 2015; Zhou et al., 2010), lack of major resistance genes in plants (Bai et al., 2018; Srinivas et al., 2019) and underdeveloped breeding technologies in certain crops can impede breeding progress (Ghag et al., 2014). Hence, the application of sterol demethylation inhibitor (DMI) azole and benzimidazole fungicides still is the most widely used method for controlling disease and limiting mycotoxin accumulation. However, the long‐term intensive use of these fungicides carries a significant risk of developing fungicide tolerance (de Chaves et al., 2022; Zhao et al., 2021), along with posing hazardous effects on both the environment and human health (Pérez‐Cantero et al., 2020; van den Bosch & Gilligan, 2008). For these reasons, time‐saving, sustainable and environmentally friendly management strategies for controlling *Fusarium* diseases are being researched and developed.

Currently, a limited number of studies have investigated the mechanisms and cellular functions of the *Fusarium* RNA interference (RNAi) pathway, a gene regulatory system that is evolutionarily conserved among eukaryotes, including fungi, that uses small RNA (sRNA) molecules for sequence‐specific transcriptional and post‐transcriptional gene silencing (Gaffar et al., 2019; Son et al., 2017). New knowledge of these mechanisms opens up the possibility of developing RNAi‐based strategies in *Fusarium* as a powerful reverse genetics tool to identify gene function at the cellular level. In the last decades, many reports provided an initial basis for a fundamental understanding of RNAi‐based strategies for disease control (Arango et al., 2024). In *Fusarium* species, some studies have used different types of RNA such as small interfering sRNA duplexes or long double‐stranded (ds)RNA to target essential genes. Inspired by the intriguing finding that plants and microbes exchange sRNAs bidirectionally to shape their interaction (known as cross‐kingdom RNAi [ckRNAi]; Weiberg et al., 2013), sRNAs or their dsRNA precursors have been used to control *Fusarium* indirectly by spray treatments (spray‐induced gene silencing [SIGS]; Koch et al., 2016) or by expressing artificial dsRNAs in the plant (known as host‐induced gene silencing [HIGS]; Koch et al., 2013). In addition, based on the discovery of RNAi in rhizospheric microorganisms, a new strategy, designated microbe‐induced gene silencing (MIGS), has been developed to protect plants from *Fusarium* infection by utilizing a beneficial microbe for sRNA delivery (Wen et al., 2023). Finally, based on the ability of mycoviruses to infect and be horizontally transmitted between fungi, a mycovirus‐based virus‐induced gene‐silencing system (VIGS) has been developed in *F. graminearum* to protect wheat from FHB under field conditions (Zhang, Wang, et al., 2023). In this review, we aim to provide an overview of the current status of RNAi signalling pathways in *Fusarium* pathogens and of RNAi‐based applications for the control of *Fusarium* diseases in crops.

## 2 RNAi PATHWAY IN *FUSARIUM* SPP.

### 2.1 RNAi pathway components in *Fusarium*

The RNAi core machinery is highly conserved in the fungal kingdom (Choi et al., 2014; Nicolás & Garre, 2016). The pathway is initiated with the cleavage of a precursor dsRNA into 21–24 nucleotide (nt) sRNA duplexes by an RNase III Dicer‐like (DCL) enzyme (Billmyre et al., 2013; Gu et al., 2023). Subsequently, the sRNA is loaded onto an Argonaute (AGO) protein to form an RNA‐induced silencing complex (RISC), where one strand of the sRNA duplex undergoes degradation. The RISC then identifies a target mRNA with complementary sequences to achieve mRNA degradation, resulting in post‐transcriptional gene silencing (PTGS; Nguyen et al., 2018). RNA‐dependent RNA polymerases (RDRPs or RDRs) are responsible for using produced small interfering (si)RNAs as primers to produce new dsRNA precursors as source for secondary siRNAs to maintain the silencing effects (Calo et al., 2012; Pak et al., 2012).

In *F. graminearum*, two *DCL* (*FgDCL1*, *FgDCL2*), two *AGO* (*FgAGO1*, *FgAGO2*) and five *RDRP* (*FgRDRP1‐5*) genes have been identified (Chen et al., 2015). Additionally, *FgQIP* (*Quelling defective 2 interacting protein*) encoding an AGO‐interacting protein and the RecQ helicase gene *FgQDE3* (*Quelling defective 3*) have been detected (Gaffar et al., 2019). To study the mechanism of the RNAi pathway, Chen et al. (2015) investigated the role of RNAi components in hairpin RNA (hpRNA)‐induced silencing of fungal genes in *F. graminearum* transformants. Deleting *FgDCL2* or *FgAGO1*, but not *FgDCL1* or *FgAGO2,* restored expression of the hpRNA‐targeted genes to the wild‐type level, indicating that *FgDCL2* and *FgAGO1* are required for hpRNA‐mediated gene silencing in *F. graminearum*. Furthermore, Gaffar et al. (2019) found that the knock‐out (KO) of *FgDCL1*, *FgDCL2*, *FgAGO1*, *FgAGO2, FgRDRP1* and *FgQIP*, but not *FgQDE3*, resulted in reduced sensitivity of the fungus to exogenous dsRNAs. These findings highlight the intricate mechanisms underlying RNAi in *F. graminearum*, but the specific roles of these genes remain elusive.

In *F. asiaticum*, two *DCL* (*FaDCL1*, *FaDCL2*), two *AGO* (*FaAGO1*, *FaAGO2*) and five *RDRP* (*FaRDRP1‐5*) genes have been predicted (Gu et al., 2023; Song et al., 2018). When the fungus was treated with dsRNAs, the transcript levels of *FaDCL2* and *FaAGO1,* but not *FaDCL1* and *FaAGO2,* were increased, suggesting that *FaDCL2* and *FaAGO1* are critical for processing of exogenous dsRNA (Song et al., 2018). In addition, the deletion of *FaDCL2* in a hpRNA transgenic strain significantly decreased the amount of fungal sRNAs, demonstrating its essential role in dsRNA cleavage (Gu et al., 2023).

The mechanism of secondary amplification of dsRNAs in fungi has not yet been well characterized and has only been described in a few fungi (Calo et al., 2012; Hammond & Keller, 2005). According to Song et al. (2018), the duration of RNA silencing in *F. asiaticum* was very transient unless exogenous dsRNAs were continuously supplied to cultures in liquid medium, suggesting a lack of secondary siRNA amplification, despite the fact that the fungus possesses five *RDRP* genes.

In addition, the FoQDE‐2 (Quelling defective 2, an AGO homologue protein) and a RDRP protein (*FolRDR1*) of *Fusarium oxysporum* f. sp. *lycopersici* were identified (Jo et al., 2018; Ouyang et al., 2023) (Table 1). However, no other RNAi pathway proteins have been reported in other *Fusarium* species. The specific roles of *DCL*, *AGO* and *RDRP* of *Fusarium* in dsRNA cleavage, siRNA recognition, mRNA degradation, as well as siRNA secondary amplification in the RNAi pathway, are still far from completely understood.

**TABLE 1:** Functions of *Fusarium* RNAi pathway genes.

Pathogen	Gene	Gene function	Reference
*F. graminearum*	*FgDCL1*	No effects on mycelial growth, environment stress responses, conidia production and virulence under the tested conditions	Chen et al. (2015); Son et al. (2017); Zeng et al. (2018)
		Pigmentation in polyethylene glycol (PEG) medium, conidiation under dimmed light in liquid synthetic nutrient (SN) culture, deoxynivalenol (DON) production on wheat spikes, virulence on wheat spikes and on detached barely and *Brachypodium distachyon* leaves	Gaffar et al. (2019)
		Sex‐specific RNAi pathway and forcible ascospore discharge	Gaffar et al. (2019); Kim et al. (2015); Son et al. (2017); Zeng et al. (2018)
		Sensitivity to exogenous double‐stranded (ds)RNA	Gaffar et al. (2019)
		Antiviral responses	Yu et al. (2018)

	*FgDCL2*	No effects on mycelial growth, environment stress responses, conidia production and virulence under the tested conditions	Chen et al. (2015); Son et al. (2017); Zeng et al. (2018)
		Pigmentation in PEG medium, conidiation under dimmed light in liquid SN culture, DON production on wheat spikes, virulence on detached barely and *B. distachyon* leaves	Gaffar et al. (2019)
		Hairpin (hp)RNA‐mediated gene silencing	Chen et al. (2015)
		Sensitivity to exogenous dsRNA	Gaffar et al. (2019)
		Antiviral responses	Yu et al. (2018)
	*FgAGO1*	No effects on mycelial growth, environment stress responses conidia production and virulence under the tested conditions	Chen et al. (2015); Son et al. (2017); Zeng et al. (2018)
		Pigmentation in PEG medium, conidiation under dimmed light in liquid SN culture, forcible ascospore discharge, DON production on wheat spikes	Gaffar et al. (2019)
		hpRNA‐mediated gene silencing	Chen et al. (2015)
		Sensitivity to exogenous dsRNA	Gaffar et al. (2019)
		Antiviral responses	Yu et al. (2018)
	*FgAGO2* (*FgSMS‐2*)	No effects on mycelial growth, environment stress responses, conidia production and virulence under the tested conditions	Chen et al. (2015); Son et al. (2017); Zeng et al. (2018)
		Conidia germination under non‐inductive conditions in SN culture, forcible ascospore discharge, DON production on wheat spikes, virulence on wheat spikes at an earlier point	Gaffar et al. (2019)
		Sex‐specific RNAi pathway, forcible ascospore discharge	Gaffar et al. (2019); Kim et al. (2015); Son et al. (2017); Zeng et al. (2018)
		Sensitivity to exogenous dsRNA	Gaffar et al. (2019)
		Antiviral responses	Yu et al. (2018)
	*FgRDRP1*	No effects on mycelial growth, environment stress responses, conidia production and virulence under the tested conditions	Chen et al. (2015)
		Pigmentation in PEG medium, forcible ascospore discharge, DON production on wheat spikes	Gaffar et al. (2019)
		Sensitivity to exogenous dsRNA	Gaffar et al. (2019)
Antiviral responses		Yu et al. (2018)

	*FgRDRP2* (*FgSAD‐1*)	No effects on mycelial growth, environment stress responses, conidia production and virulence under the tested conditions	Chen et al. (2015)
		Pigmentation in PEG medium, conidiation under dimmed light in liquid SN culture, DON production on wheat spikes	Gaffar et al. (2019)
		Forcible ascospore discharge	Gaffar et al. (2019); Son et al. (2011)
	*FgRDRP3*	No effects on mycelial growth, environment stress responses, conidia production and virulence under the tested conditions	Chen et al. (2015)
		Pigmentation in PEG medium, conidiation under dimmed light in liquid SN culture, conidia germination under non‐inductive conditions, ascospore germination; DON production on wheat spikes	Gaffar et al. (2019)
	*FgRDRP4*	No effects on mycelial growth, environment stress responses, conidia production and virulence under the tested conditions	Chen et al. (2015)
		Pigmentation in PEG medium, conidiation under dimmed light in liquid SN culture, conidia germination but with multiple germ tubes under non‐inductive conditions, ascospore germination, DON production on wheat spikes	Gaffar et al. (2019)
		Antiviral responses	Yu et al. (2018)
	*FgRDRP5*	No effects on mycelial growth, environment stress responses, conidia production and virulence under the tested conditions	Chen et al. (2015)
	*FgQIP*	Pigmentation in PEG medium, conidiation under dimmed light in liquid SN culture, forcible ascospore discharge, DON production on wheat spikes	Gaffar et al. (2019)
		Sensitivity to exogenous dsRNA	Gaffar et al. (2019)
*FgQDE3*	Pigmentation in PEG medium, conidiation under dimmed light in liquid SN culture, forcible ascospore discharge, DON production on wheat spikes	Gaffar et al. (2019)
*F. asiaticum*	*FaAGO1*	dsRNA‐mediated gene silencing	Song et al. (2018)
	*FaAGO2*	Not involved in dsRNA‐mediated gene silencing	Song et al. (2018)
	*FaDCL1*	Not involved in dsRNA‐mediated gene silencing	Song et al. (2018)
	*FaDCL2*	dsRNA‐mediated gene silencing	Song et al. (2018)
		RNA‐based antiviral defence	Gu et al. (2023)
	*FaRDRP1, FaRDRP2, FaRDRP3, FaRDRP4, FaRDRP5*	Not involved in mycelial growth, colony morphology, asexual development, sexual development and secondary small interfering (si)RNA amplification	Song et al. (2018)
*F. oxysporum* f. sp. *lycopersici*	*FolRDR1*	Mycelial growth, conidia production, virulence on tomato	Ouyang et al. (2023)
	*FoQDE‐2*	Virulence on tomato	Jo et al. (2018)

### 2.2 Biological processes regulated by RNAi in *Fusarium*

The RNAi pathway of filamentous fungi plays roles in antiviral defence, genomic stability, heterochromatin formation, silencing of transposable elements, environmental stress response, development and pathogenicity by using sRNAs (Chang et al., 2012; Lax et al., 2020). Even though the mechanism of the RNAi pathway in *Fusarium* to produce sRNAs is not completely understood, several biological processes have been elucidated to be regulated by RNAi.

In *F. graminearum*, the RNAi pathway is employed as a defence against mycoviruses. The expression of RNAi pathway genes is induced by specific viruses. In mutants lacking or overexpressing RNAi genes, viral RNA accumulation and virus‐derived siRNA levels changed depending on the specific gene and virus (Yu et al., 2018). Early research suggested that KO mutants of *F. graminearum DCL*, *AGO* and *RDRP* genes did not show any defects in mycelial growth, environmental stress responses, conidia production as well as the virulence on flowering wheat heads, tomato fruits and maize silk under their experimental conditions (Chen et al., 2015; Zeng et al., 2018). However, a subsequent report showed that under different experimental conditions, these genes participate in synthesizing mycelial pigments, asexual development and DON production on wheat spikes to varying degrees. More importantly, single KO mutants of *FgDCL1* and *FgAGO2* showed compromised FHB development at an earlier stage on the wheat spikes (Gaffar et al., 2019). Furthermore, as reported by Werner et al. (2021), *F. graminearum* secretes DCL‐dependent sRNAs for targeting plant host defence genes to promote pathogenesis. In this report, KO of either *FgDCL1* or *FgDCL2* resulted in smaller necrotic lesions on detached *Brachypodium distachyon* and barley leaves.

*Fusarium graminearum* possesses a functional meiotic silencing of unpaired DNA (MSUD) mechanism; this sex‐induced RNAi mechanism uses sRNA‐mediated RNAi for ascosporogenesis, canonical RNAi pathway genes are involved in this mechanism (Son et al., 2011, 2017). During the sexual stage, *FgDCL1*, *FgDCL2* and *FgAGO2* (*FgSMS‐2*) expression increases after sexual induction (Son et al., 2017; Zeng et al., 2018). Single KO mutants of *FgDCL1* and *FgAGO2*, double KO mutants of *FgDCL1* and *FgDCL2* and double KO mutants of *FgAGO1* and *FgAGO2* all exhibit significant impairments in forcible ascospore discharge (Gaffar et al., 2019; Kim et al., 2015; Son et al., 2017; Zeng et al., 2018). Furthermore, KO mutants of *RDRP* genes *FgRDRP1‐4* display defects in both forcible ascospore discharge and germination (Chen et al., 2015; Gaffar et al., 2019; Son et al., 2011) (Table 1).

In *F. asiaticum*, *FaDCL2* has been identified to play a critical role in RNA‐based antiviral defence but not in growth or asexual conidiation (Gu et al., 2023), while *FaRDRP1‐5* is not involved in mycelial growth, asexual or sexual development (Song et al., 2018). In *F. oxysporum* f. sp. *lycopersici*, an AGO homologue protein gene, *FolQDE‐2*, is involved in virulence in tomato but not vegetative growth (Jo et al., 2018). By contrast, KO of *FolRDRP1* not only led to dramatically decreased penetration on tomato but also lower sporulation and abnormal conidia (Ouyang et al., 2023) (Table 1).

Overall, these findings highlight that the RNAi pathway components of different *Fusarium* species play diverse roles in various cell processes, including antiviral defence, fungal development and virulence. Understanding how the RNAi pathway regulates these biological processes is helpful for fully comprehending the RNAi pathway in *Fusarium*.

## 3 HARNESSING THE RNAi PATHWAY IN *FUSARIUM*

### 3.1 Expressing endogenous dsRNA for gene function analysis in fungal cells

**FIGURE 1:**
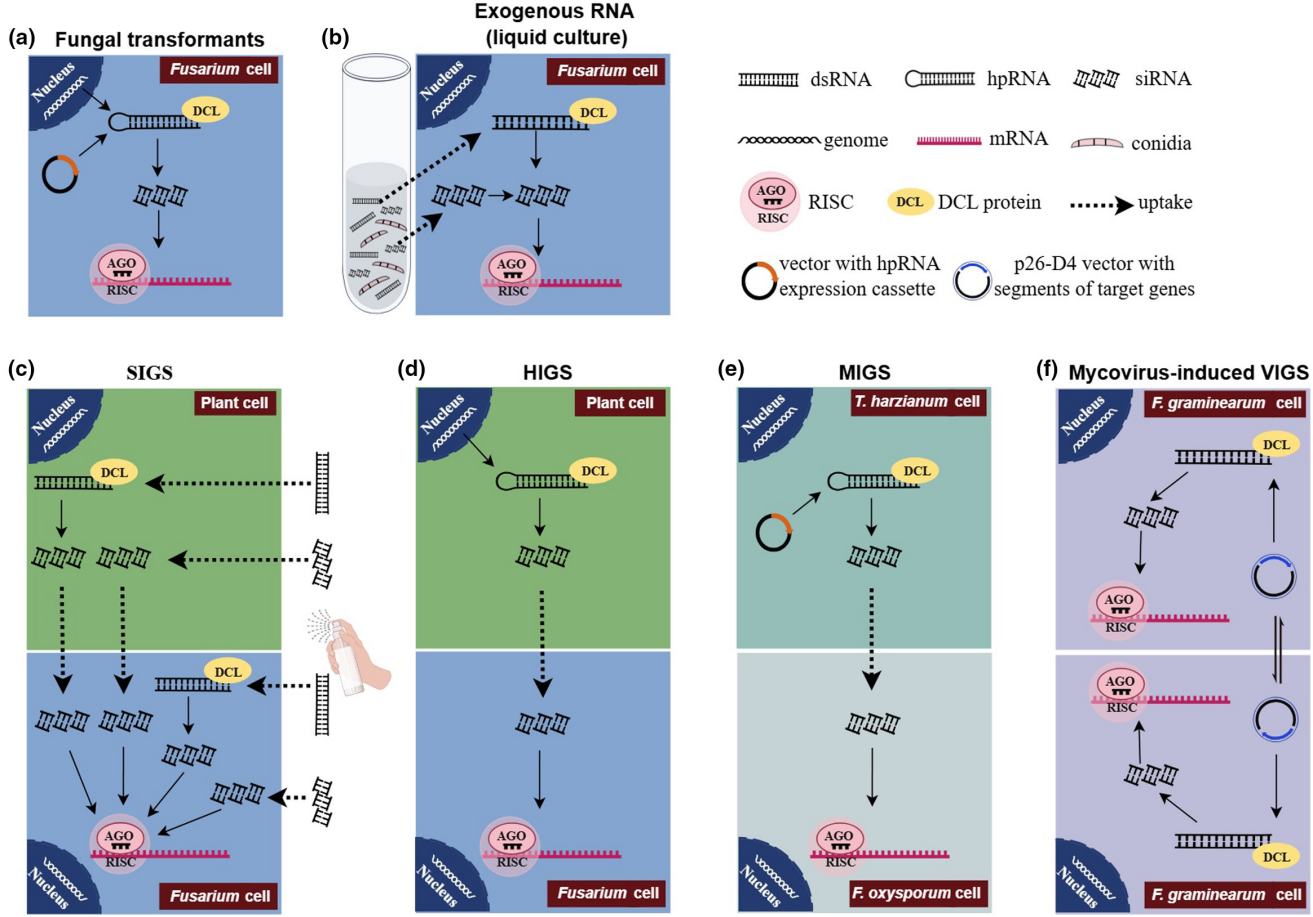
Schematic overview of RNAi applications in *Fusarium*. (a) RNAi in fungal cells: Endogenous hairpin (hp)RNAs expressed in *Fusarium* cells are cleaved into small interfering (si)RNAs by DCL proteins. One strand of these siRNA duplexes is then loaded onto an RNA‐induced silencing complex (RISC) and guides the degradation of target transcripts. (b) RNAi in liquid medium: Exogenous dsRNAs or siRNAs are taken up by *Fusarium* cells, subsequently pass through the fungal RNAi pathway. (c) Spray‐induced gene silencing (SIGS): Exogenously applied dsRNA/siRNA on the plant surface may transit through plant cells or be directly taken up by *Fusarium* cells, triggering the silencing of fungal genes. (d) Host‐induced gene silencing (HIGS): The siRNAs processed from hpRNAs expressed in transgenic plants can be internalized by infecting *Fusarium* cells and pass through the fungal RNAi pathway. (e) MIGS: The siRNA processed from dsRNA expressed by engineered microbes such as *Trichoderma harzianum* can be internalized by *Fusarium* (such as *F. oxysporum*) cells and go through the fungal RNAi pathway. (f) Mycovirus‐based virus‐induced gene‐silencing (VIGS): FgGMTV1‐based VIGS vector p26‐D4 with segments of target genes triggers the fungal RNAi pathway and can be transmitted horizontally from the hypovirulent strains to virulent strains. Detailed explanations are found in the main text. The figure was created by Figdraw (www.figdraw.com).

RNAi‐based approaches for reverse genetic analysis of fungal gene functions in *Fusarium* have found wide application (Table 2). Endogenous dsRNAs (typically hpRNA) are expressed in *Fusarium* cells either from a plasmid or from the integrated genome carrying an RNAi construct, thereby triggering the silencing of target genes (Chen et al., 2015; Song et al., 2018) (Figure 1a). As early as 2005, McDonald et al. (2005) showed the importance of the transcriptional regulator gene *TRI6*, which is involved in DON biosynthesis, for fungal virulence and disease symptom development by transforming *F. graminearum* with an inverted repeat construct forming hpRNAs targeting *TRI6*. Moreover, Chen et al. (2015) used transformants carrying a sequence‐specific hpRNA cassette to study the function of the 14‐α demethylase *FgCYP51A*; *FgPKS12*, involved in pink pigment biosynthesis; and the Calcineurin regulatory subunit B *FgCNB1*. The transformants exhibited increased sensitivity to DMI fungicides, showed white colony morphology and were defective in growth, similar to their KO mutants. As summarized in Table 2, the functions of many *Fusarium* genes have since been explored using a gene silencing strategy, including *TRI6* of *F. culmorum* (Scherm et al., 2011); three MAP kinase signalling genes (*FMK1*, *HOG1* and *PBS2*), *FOW2* (a putative transcription regulator), *PEX6* (*Peroxisomal biogenesis factor 6*) along with four fasciclin‐like protein genes (*FoFLP1*, *FoFLP3*, *FoFLP4* and *FoFLP5*) of *F. oxysporum* f. sp. *lycopersici* (Chauhan & Rajam, 2022; Pareek & Rajam, 2017; Shanmugam et al., 2017; Tetorya & Rajam, 2018); *SGE1* (*SIX Gene Expression 1*) of *F. oxysporum* f. sp. *cubens* (Fernandes et al., 2016), *CYP51* (*P450 lanosterol C‐14 α‐demethylase*), *CHS1* (*Chitin synthase 1*) and *EF2* (*Elongation factor 2*) of *F. oxysporum* f. sp. *radicis‐lycopersici* (Mosa & Youssef, 2021); *FKS1* (*1,3‐D‐glucan synthase*), *CSN1* (*Chitosanase*) and *CHSV* (*Chitin synthase*) of *F. solani* (Ha et al., 2006; Liu et al., 2010; Shanmugam et al., 2017); and fumonisin biosynthesis genes *FUM1* and *FUM8* of *F. verticillioides* (Johnson et al., 2018). Silencing of these genes resulted in numerous defects, including but not limited to growth, virulence and mycotoxin production.

**TABLE 2:** List of *Fusarium* genes targeted through RNAi.

Pathogen	Target gene	RNAi application	RNA	Phenotype	Reference
*F. verticillioides*	*GUS* reporter	HIGS^a^ (tobacco)	hpRNA	*GUS* silencing	Tinoco et al. (2010)
	*FUM1*, *FUM8*	Transformants^b^	hpRNA	Reduced fumonisin	Johnson et al. (2018)
*F. solani*	*FsFKS1*	Transformants	hpRNA	Reduced conidia germination	Ha et al. (2006)
	*CSN1*	Transformants	hpRNA	Reduced chitosanase activity, enhanced virulence on pea	Liu et al. (2010)
	*CHSV*	Transformants	hpRNA	Reduced conidia production, lower virulence on tomato	Shanmugam et al. (2017)
*F. graminearum*	*CYP51A*, *CYP51B*, *CYP51C*	Liquid culture^c^	dsRNA	Mycelial growth defects, inhibited fungicide tolerance	Koch et al. (2013)
		HIGS (*Arabidopsis*, barley detached leaves, *Brachypodium distachyon* spikes)	hpRNA	Enhanced plant resistance	Koch et al. (2013); He et al. (2019); Werner et al. (2020); Höfle et al. (2020)
		SIGS (detached barley leaves)^d^	dsRNA	Reduced disease development	Koch et al. (2016); Koch et al. (2019); Höfle et al. (2020); Kim et al. (2023)
		SIGS (detached barley leaves)	siRNA	Reduced disease development	Koch et al. (2016)
	*FgPKS12*	Transformants	hpRNA	Mycelial growth defects	Chen et al. (2015)
	*FgCNB1*	Transformants	hpRNA	Mycelial growth defects	Chen et al. (2015)

	*FgCYP51A‐FgPKS12‐FgTRI6*	Transformants	hpRNA	Mycelial growth defects, reduced deoxynivalenol (DON) production	Chen et al. (2015)
	*TRI6*	Transformants	hpRNA	Reduced DON production, lower virulence on wheat and barley	McDonald et al. (2005); Baldwin et al. (2018)
		Liquid culture	dsRNA	No effect on DON production	Hao et al. (2021)
		SIGS (detached/intact wheat spikes)	dsRNA	Inhibited disease development on detached spikes and on intact spikes under greenhouse conditions but not in growth chamber conditions	Hao et al. (2021)
		HIGS (barley)	hpRNA	No effect on plant resistance and DON production	Gao et al. (2024)
	*CHS3b*	Transformants	hpRNA	Mycelial growth defects, increased stress sensitivity, decreased chitin biosynthesis	Cheng et al. (2015)
		HIGS (wheat spikes/seedlings)	hpRNA	Enhanced plant resistance, inhibited DON production	Cheng et al. (2015)
	*Fg00677*, *Fg08731*, *CYP51A‐CYP51B‐CYP51C*	HIGS (*B. distachyon* spikes)	hpRNA	Enhanced plant resistance	He et al. (2019)
	*FgSGE1‐FgSTE12‐FgPP1*	Transformants	hpRNA	Mycelial growth defects, reduced DON production, lower virulence on wheat spikes	Wang et al. (2020)
		HIGS (wheat spikes)	hpRNA	Enhanced plant resistance, inhibited DON production	Wang et al. (2020)
	*FgAGO1*, *FgAGO2*, *FgDCL1*, *FgDCL2*	SIGS (detached barley leaves)	dsRNA	Reduced disease development	Werner et al. (2020)
	*CHS7*, *GLS*, *PKC*	Transformants	hpRNA	Mycelial growth defects, lower virulence on wheat	Yang et al. (2021)
		Liquid culture	dsRNA	Reduced mycelial growth	Yang et al. (2021)
		SIGS (wheat spikes/detached leaves)	dsRNA	Inhibited DON production, reduced disease development	Yang et al. (2021)
*Fg10360*, *Fg13150*, *Fg06123*	SIGS (detached barley leaves)	dsRNA	Reduced disease development	Kim et al. (2023)

	*FgPMA1*	Transformants	hpRNA	Mycelial growth defects, reduced conidia germination, reduced DON production, lower virulence on wheat	Wu et al. (2023)
		Liquid culture	dsRNA	Inhibited mycelial growth, inhibited conidia production	Wu et al. (2023)
		SIGS (wheat spikes)	dsRNA	Inhibited disease development	Wu et al. (2023)
	*GPMK1*, *FgCHY1*, *FgSR*, *TRI5*, *FgTEAA*	HIGS (detached wheat leaves, intact wheat spikes/seedlings)	hpRNA	Enhanced plant resistance, inhibited DON production	Shuai et al. (2024)
		SIGS (detached wheat leaves)	siRNA	Reduced disease development	Shuai et al. (2024)
*TRI1*, *TRI5*, *TRI10*, *TRI101*, *FgP1*, *FgPP1*, *FgCYP51C*	Mycovirus‐based VIGS (wheat spikes)^e^	hpRNA	Reduced disease development, inhibited DON production	Zhang, Shi, et al. (2023)
*F. oxysporum*	*CYP51B*	HIGS (soybean)	hpRNA	Enhanced plant resistance	Pérez et al. (2021)
	*FoPMT2*	MIGS (solid culture)^f^	hpRNA	Inhibited mycelial growth	Wen et al. (2023)
		MIGS (rice)	hpRNA	Reduced disease development	Wen et al. (2023)
*F. oxysporum* f. sp. *cubense*	*VEL*, *FTF1*	Liquid culture	siRNA	Reduced mycelial growth, reduced conidia production	Ghag et al. (2014)
		HIGS (banana)	hpRNA	Enhanced plant resistance	Ghag et al. (2014)
	*ERG6*, *ERG11*	Liquid culture	dsRNA	Inhibited fungicide tolerance	Dou et al. (2020)
		HIGS (banana)	hpRNA	Enhanced plant resistance	Dou et al. (2020)
	*SGE1*	Transformants	hpRNA	Reduced conidia production, reduced virulence on banana	Fernandes et al. (2016)
	14 genes involved in conidia germination	Liquid culture	dsRNA	Inhibited conidia germination	Mumbanza et al. (2013)
*F. oxysporum* f. sp. *conglutinans*	*FRP1*, *OPR*, *FOW2*	HIGS (*Arabidopsis*)	hpRNA	Enhanced plant resistance	Hu et al. (2015)
*F. oxysporum* f. sp. *lycopersici*	*FMK1*, *HOG1*, *PBS2*	Transformants	hpRNA	Altered conidial morphology, lower virulence on tomato	Pareek and Rajam (2017)
	*FOW2*	Transformants	hpRNA	Mycelial growth defects, reduced spores production, lower virulence on tomato	Shanmugam et al. (2017)
		HIGS (tomato/*Arabidopsis*)	hpRNA	Enhanced plant resistance	Bharti et al. (2017)
	*CHSV*	HIGS (tomato)	hpRNA	Enhanced plant resistance	Bharti et al. (2017)
	*FolRDR1*	Liquid culture	dsRNA	Reduced conidia production	Ouyang et al. (2023)
		SIGS (tomato seedlings)	dsRNA	Inhibited disease development	Ouyang et al. (2023)
	*ODC*	HIGS (tomato)	hpRNA	Enhanced plant resistance	Singh et al. (2020)
	*PEX6*	Transformants	hpRNA	Mycelial growth defects, reduced conidia production, lower virulence on tomato	Tetorya and Rajam (2018)
		HIGS (tomato)	hpRNA	Enhanced plant resistance	Tetorya and Rajam (2021)
	*GAS1*	HIGS (tomato)	hpRNA	Enhanced plant resistance	Tetorya & Rajam, 2021
	*FoFLP3*	Transformants	hpRNA	Reduced conidia production, lower virulence on tomato	Chauhan and Rajam (2022)
	*FoFLP1*, *FoFLP4*, *FoFLP5*	Transformants	hpRNA	Reduced conidia production, lower virulence on tomato	Chauhan and Rajam (2022)
		HIGS (tomato)	hpRNA	Enhanced plant resistance	Chauhan and Rajam (2024)
*F. oxysporum* f. sp. *radicis‐lycopersici*	*CYP51*, *CHS1*, *EF2*	Transformants	hpRNA	Lower virulence on tomato	Mosa and Youssef (2021)
	*CYP51‐CHS1‐EF2*	Liquid culture	hpRNA	Inhibited mycelial growth	Mosa and Youssef (2021)
		SIGS (tomato seedlings)	dsRNA	Inhibited disease development	Mosa and Youssef (2021)
*F. asiaticum*	*MYO5*	Transformants	hpRNA	Mycelial growth defects, increased cell wall stress sensitivity, reduced conidia production, reduced perithecia production, lower virulence on wheat	Song et al. (2018)
		SIGS (wounded wheat coleoptile)	dsRNA	Reduced disease development	Song et al. (2018)
	*β2‐Tubulin*	Transformants	hpRNA	Mycelial growth defects, reduced conidia production, increased fungicide sensitivity, lower virulence on wheat	Gu et al. (2019)
		Liquid culture	dsRNA	Inhibited conidia production, inhibited mycelial growth	Gu et al. (2019)
		SIGS (wounded wheat coleoptile)	dsRNA	Reduced mycelial growth, reduced disease development	Gu et al. (2019)
*F. culmorum*	*TRI6*	Transformants	hpRNA	Reduced DON production, lower virulence on wheat	Scherm et al. (2011)
	*FcGLS1*, *FcFMK1*, *FcCHSV*	HIGS (wheat leaves/spikes)	hpRNA	Enhanced plant resistance	Chen et al. (2016)
	*FcCYP51A, FcCYP51B, FcCYP51C*	Liquid culture	dsRNA	Inhibited mycelial growth	Koch et al. (2018)
	*TRI5*	Liquid culture	dsRNA	Inhibited DON production	Tretiakova et al. (2022)
		SIGS (wheat spikes/detached leaves)	dsRNA	Reduced disease development	Tretiakova et al. (2022)

^a^ HIGS, host‐induced gene silencing: hairpin (hp)RNA or double‐stranded (ds)RNA expression in the plant host.

^b^ Transformants: expression of hpRNA in fungal cells.

^c^ Treatment of fungal liquid cultures with exogenous dsRNA/small interfering (si)RNA.

^d^ SIGS, spray‐induced gene silencing: treatment of plants with exogenous dsRNA/siRNA.

^e^ Mycovirus‐induced virus‐induced gene silencing (VIGS): treatment with a modified mycovirus resulting in a hypovirulent fungus due to gene silencing.

^f^ MIGS, microbe‐induced gene sielncing: use of a transgenic beneficial fungus to induce gene silencing in a target fungus.

The expression of dsRNA constructs targeting different segments of a gene can lead to different levels of silencing. In *F. graminearum*, the chitin synthase FgCHS3b is essential for survival and colonization during plant infection. Cheng et al. (2015) found that three (CHS3b‐1, ‐3 and ‐5) out of five RNAi constructs corresponding to different gene segments exhibited the most effective silencing, resulting in abnormal mycelial growth, diminished chitin biosynthesis and impaired virulence. In another study, Yang et al. (2021) identified the most effective regions of three *F. graminearum* genes (*FgCHS7, FgGLS* and *FgPKC*) for silencing. Chitin synthase CHS7 is required for mycelial growth, perithecia formation and pathogenicity, while the glucan synthase GLS and the kinase C PKC are crucial for fungal survival. As a result, four out of seven segments of *CHS7*, three out of 13 segments of *GLS* and one out of eight segments of *PKC* effectively silenced their respective genes. In a recent study, Wu et al. (2023) demonstrated that the plasma membrane H^+^‐ATPase FgPMA1 of *F. graminearum* regulates fungal development, pathogenicity and sensitivity to the fungicide phenamacril. Three (FgPMA1RNAi‐1, ‐2, and ‐5) out of six RNAi constructs corresponding to different gene segments showed effective silencing, leading to impaired fungal development and pathogenicity on detached wheat leaves, coleoptiles and wheat spikes. In *F. asiaticum*, *MYO5* encodes a class I myosin that is independent of the regulation of eukaryotic cytokinesis, organelle transport, cell polarization and sensitivity to the fungicide phenamacril, and loss of its gene function is lethal in *F. graminearum* (Zhang et al., 2015; Zheng et al., 2015). Five out of eight RNAi constructs corresponding to different gene segments showed the ability to silence *MYO5*, with the MYO5‐8 segment showing the highest silencing efficiency (Song et al., 2018). Another gene, *β2‐Tubulin*, is important for the maintenance of fungal development and resistance to the fungicide carbendazim in *Fusarium* species (Liu et al., 2013; Qiu et al., 2012). In *F. asiaticum*, *β2‐Tubulin* was divided into four segments to generate dsRNA constructs. Among them, the Faβ2TUB‐3 segment showed the highest efficiency in inhibiting conidia development, mycelial growth, fungicide resistance and pathogenicity (Gu et al., 2019).

However, a mechanistic explanation of why different regions led to different RNAi efficiency in fungal cells is not yet sufficiently clear. The base composition of the dsRNA sequence is probably one determining factor. For instance, in *Paramecium*, DCL2 has a sequence preference for 5′ U and 5′ AGA, DCL3 has a preference for 5′ UNG, while DCL5 has a preference for 5′ UAG and 3′ CUAC/UN, where N stands for any nucleotide (nt) (Hoehener et al., 2018). However, it is unclear whether such sequence preferences exist for fungal DCLs. Another possibility is the selectivity of AGOs for a 5′ nt in sRNAs. While there are no direct studies on the 5′ nt preference of *Fusarium* AGOs, the general understanding of Argonaute function in the model fungus *Neurospora crassa* suggests a likely preference for 5′ U (Lee et al., 2010). While the selection of active siRNAs is generally based on the identification of siRNAs with specific sequence and structural properties, the efficiency of RNAi is also dependent on the structure of the target mRNA. The accessibility of certain local target structures on the mRNA is an important determinant of siRNA‐mediated RNAi. For example, transfection of HeLa and HepG2 cells showed that siRNAs targeting regions of mRNA thought to have unpaired 5′ and 3′ ends resulted in greater gene silencing than regions thought to have other types of secondary structures (Gredell et al., 2008). Overall, a combination of sequence preference in conjunction with DCL cleavage, AGO loading and the structure of target mRNAs may contribute to variable RNAi efficiency in fungi.

Interestingly, a single dsRNA construct can target multiple genes simultaneously by carrying segments from different genes (Chen et al., 2015). Transgenic *F. graminearum* containing a dsRNA construct with a tandem DNA fragment *FgCYP51A*‐*FgPKS12*‐*FgTRI6* exhibited several phenotypes similar to these of their knock‐down (KD) or KO mutants. In a later report, Wang et al. (2020) selected the three target genes *FgSGE1*, *FgSTE12* and *FgPP1* to produce the chimeric RNAi construct FgSGE1‐STE12‐PP1. *FgSGE1* (a *WOR1* orthologue) is required for conidia formation and sexual reproduction, pathogenicity and DON accumulation (Jonkers et al., 2012), while the transcription factor *FgSTE12* is involved in perithecia formation (Gu et al., 2015) and the phosphatase *FgPP1* is essential for fungal survival (Yun et al., 2015). *F. graminearum* carrying FgSGE1‐STE12‐PP1 showed multiphenotypic changes similar to their KO mutants. These findings provide a reliable basis for simultaneously silencing multiple genes to affect various aspects of fungal development and virulence (Table 2). In summary, endogenously expressed dsRNA in *Fusarium* can trigger the fungal RNAi signalling pathway to silence one or more target genes, and this is a straightforward approach for screening target gene functions and searching for target genes suitable for RNAi‐based disease resistance strategies.

### 3.2 Application of antifungal exogenous dsRNA to *Fusarium* cultures

Several studies suggest that the direct application of exogenous dsRNAs, including sRNAs duplexes triggers gene silencing in *Fusarium* (Table 2). In vitro treatment of axenic and liquid fungal cultures with a 791‐nt stacked dsRNA (CYP3‐dsRNA) targeting three P450 lanosterol C‐14 α‐demethylases genes (*FgCYP51A*, *FgCYP51B* and *FgCYP51C*) inhibited mycelial growth and induced abnormal branching both in *F. graminearum* and *F. culmorum* (Koch et al., 2013, 2018). Similarly, exogenous dsRNAs CHS7‐4, GLS‐6 and PKC‐5 also strongly inhibited mycelial growth in *F. graminearum* (Yang et al., 2021). In *F. oxysporum* f. sp. *cubense*, dsRNAs derived from 14 genes exhibited varying degrees of inhibition of conidia germination in a liquid culture (Mumbanza et al., 2013) and stacked dsRNA constructs corresponding to two C‐24 sterol methyltransferase genes *ERG6A* and *ERG6B* or three *CYP51* homologues *EGR11A*, *EGR11B* and *EGR11C* also showed fungistatic effects (Dou et al., 2020). Moreover, FolRDR1‐dsRNA1 and FolRDR1‐dsRNA2 directed against *FolRDR1* of *F. oxysporum* f. sp. *lycopersici* cultures effectively inhibited conidia production (Ouyang et al., 2023). In addition, the accumulation of DON produced by *F. culmorum* in trichothecene‐inducing liquid medium was inhibited by dsRNAs targeting trichodiene synthase *TRI5* (Tretiakova et al., 2022).

In addition to long dsRNAs, exogenous siRNAs similarly inhibit mycelial growth in fungal cultures, rendering the treatment independent of DCL activity in the target fungus (Wang et al., 2016; Werner et al., 2020). Ghag et al. (2014) investigated the effects of exogenous siRNAs targeting the two genes *velvet* (*VEL*) and *Fusarium* transcription factor 1 (*FTF1*) from *F. oxysporum* f. sp. *cubense*. *VEL*, which is highly conserved in the *Fusarium* genus, is involved in the regulation of sexual and asexual development, secondary metabolism and virulence in *F. graminearum* (Jiang et al., 2011). *FTF1* encodes a Zn(II)2–Cys6 binuclear DNA‐binding protein; it is strongly upregulated during the infection of beans by *F. oxysporum* f. sp. *phaseoli* (de Vega‐Bartol et al., 2011). Consistent with their roles in related *Fusarium* species, exogenous siRNAs hindered the mycelial growth of *F. oxysporum* f. sp. *cubense*.

Based on the degree of sequence conservation with the targeted region and the function of targeted genes in different fungal species, sequences of certain genes can be selected for developing broad‐spectrum antifungal activity in RNAi applications. For instance, dsRNAs of MYO5‐8 derived from *F. asiaticum* also inhibited mycelial growth and induced abnormal mycelium morphology in cultures of *F. graminearum*, *F. tricinctum* and *F. oxysporum* f. sp. *lycopersici* (Song et al., 2018). Similarly, Faβ2TUB‐3 dsRNAs triggered mycelium growth defects not only in *Fusarium* species such as *F. graminearum*, *F. tricinctum*, *F. oxysporum* and *F. fujikuroi* but also other pathogenic fungi such as *Botrytis cinerea*, *Magnaporthe oryzae* and *Colletotrichum truncatum* (Gu et al., 2019). Finally, FgPMA1RNAi‐5‐dsRNA derived from *F. graminearum* inhibited mycelial growth and conidia production both in *F. graminearum* and *F. asiaticum* (Wu et al., 2023).

These findings confirm the capability of *Fusarium* mycelia to internalize exogenous dsRNA/siRNA molecules from the environment (Figure 1b). However, the molecular mechanisms used by *Fusarium* pathogens to internalize dsRNAs/siRNAs remain unclear, although it is plausible, but not proven, that clathrin‐mediated endocytosine might be involved (Šečić & Kogel, 2021; Wytinck et al., 2020).

### 3.3 Control of *Fusarium* infections by SIGS

In SIGS, exogenous sequence‐specific dsRNAs/siRNAs are directly applied to plants to control pests and diseases plants (Cai et al., 2018). These RNAs can be absorbed by plant cells and processed into siRNAs, or taken up by fungi from the plant surface and cleaved into siRNAs by fungal cells (Koch et al., 2016; Wang et al., 2016) (Figure 1c). SIGS does not require the production of transgenes, which saves time and money and is also more likely to be accepted by consumers (Machado et al., 2018). dsRNAs can be effective even at low doses, and as the cost of dsRNA synthesis has fallen in recent years, the method has great agronomic potential (Hough et al., 2022; Machado et al., 2018; Taning et al., 2020).

In recent years, the fast development of SIGS has also benefited the control of *Fusarium* (Table 2). Koch et al. (2016) showed for the first time that spraying 791 nt CYP3‐dsRNA and siRNAs derived from *F. graminearum CYP51* genes on detached barley leaves prior to fungal inoculation can effectively inhibit fungal infections, suggesting that SIGS could be an option for *Fusarium* control. In the follow‐up studies, SIGS targeting single or double *CYP51* genes likewise decreased disease symptoms on detached barley leaves (Höfle et al., 2020; Koch et al., 2019). Intriguingly, components of the RNAi pathway have also been identified as SIGS targets against *F. graminearum*. Pairwise spraying of computationally or manually designed dsRNAs against *FgAGO1*, *FgAGO2*, *FgDCL1* and *FgDCL2* can inhibit infection symptoms of *F. graminearum* on detached barley leaves (Werner et al., 2020). However, this finding is at first glance not consistent with earlier reports showing that *FgDCL2* and *FgAGO1* are required for hpRNA‐mediated gene silencing in fungal cells (Chen et al., 2015). Possible explanations for this discrepancy have been proposed by Mann et al. (2023), namely (1) unknown off‐targets from the dsRNA targeting fungal *DCL* genes could be influencing virulence whereby off‐targets involve a DCL‐independent gene silencing pathways, (2) the incomplete downregulation of the DCL‐dependent gene silencing pathways by homologous dsRNA may be more impactful on fungal virulence and growth than the complete KO of the same pathway, (3) mycovirus infection of *Fusarium* may affect the results in different laboratories, and (4) the complexity of the plant and pathogen interaction. Recently, Kim et al. (2023) reported that silencing 13 genes in *F. graminearum* resulted in various mycelium growth defects. However, only three of these genes, namely *Fg10360*, *Fg13150* and *Fg06123*, with unclear molecular functions, have shown potential for SIGS on detached barley leaves. This result underscores that not all mycelium growth‐related genes prove amenable to SIGS, highlighting the importance of discerning suitable targets.

Up to now, several genes have been identified as suitable SIGS targets for managing *Fusarium* in wheat (*Triticum aestivum*). In *F. graminearum*, dsRNAs CHS7‐4, GLS‐6, PKC‐5 and FgPMA1RNAi‐1, ‐2, and ‐5 showed strong silencing efficiency on detached wheat leaves or spikes (Wu et al., 2023; Yang et al., 2021). Spraying exogenous siRNAs targeting the five genes *TRI5*, *GPMK1*(a kinase protein), *FgCHY1*(a zinc finger protein), *FgSR* (a transcription factor) and *FgTEAA* (a cell‐end marker protein), either separately or in combination, resulted in a notable reduction in *Fusarium* infection symptoms (Shuai et al., 2024). Application of MYO5‐8 and Faβ2TUB‐3 dsRNA on wounded wheat coleoptiles increased resistance to *F. asiaticum* (Song et al., 2018). Interestingly, Faβ2TUB‐3 dsRNA not only conferred resistance of wheat coleoptiles to *F. asiaticum*, but also enhanced the resistance to *B. cinerea* on cucumber, *M. oryzae* on barley and *C. truncatum* on soybean (Gu et al., 2019). In addition, spraying TRI5‐dsRNA on detached wheat leaves and flowering heads efficiently decreased *F. culmorum* disease symptoms (Tretiakova et al., 2022).

So far, four target genes have been used for controlling *F. oxysporum* on tomato. In *F. oxysporum* f. sp. *radicis‐lycopersici*, SIGS targeting *FoCYP51*, *FoCHS1* and *FoEF2* improved resistance against infection (Mosa & Youssef, 2021). In *F. oxysporum* f. sp. *lycopersici*, spray application of FolRDR1‐dsRNA1 and FolRDR1‐dsRNA2 on pre‐infected leaves led to a significant reduction in symptoms of tomato wilt disease (Ouyang et al., 2023).

Although these studies have identified some promising target genes and demonstrated the potential of SIGS in controlling *Fusarium* diseases, all of them were processed in laboratory settings where experimental conditions can be precisely controlled. It is worth mentioning that, despite previous findings that suppression of *F. graminearum TRI6* expression reduced DON production and pathogenicity in fungal cells by endogenous dsRNA (Baldwin et al., 2018; McDonald et al., 2005), adding TRI6‐dsRNA to cultures in toxin induction medium did not affect DON production (Hao et al., 2021). When the same TRI6‐dsRNA was applied to intact spikes, it significantly reduced FHB and DON under greenhouse conditions, but failed to prevent FHB spread in growth chamber conditions (Hao et al., 2021). These studies show that the ultimate efficiency of RNAi, even when targeting the same gene, is the result of different conditions with multiple interacting factors. In open field conditions, the growth status of plants and environmental factors are much more complex than in the laboratory. To our best knowledge, there is no case of success using SIGS to control *Fusarium* in field yet. Therefore, in addition to identifying the most suitable target genes, many other factors need to be considered (Das & Sherif, 2020; Hoang et al., 2022).

### 3.4 Control of *Fusarium* infections by HIGS

HIGS is a transgene‐mediated technique (Nowara et al., 2010). The dsRNAs, typically hpRNA or dsRNA produced from inverted promoters, are produced from engineered plant genomes and subsequently processed into siRNAs. When the pathogen infects the plant, siRNAs can be transported from a host cell into the pathogen cell and specifically target mRNA of fungal target genes (Figure 1d) (Ghag et al., 2014; Koch et al., 2013; Wang et al., 2016). This discovery showed that sRNA can be transferred between interacting organisms, providing the first evidence of the ckRNAi mechanism.

In 2010, Tinoco et al. first demonstrated that a *GUS* (*ß‐glucuronidase*) hairpin interfering cassette expressed in tobacco leaves specifically downregulated the *GUS* reporter gene in *F. verticillioides*. Since then, many studies confirmed that HIGS can be used to enhance crop resistance against *Fusarium* species (Table 2). The use of HIGS to control *Fusarium* was first demonstrated by Koch et al. (2013). Transgenic *Arabidopsis thaliana* and barley plants carrying the CYP3‐dsRNA cassette showed significantly improved resistance to *F. graminearum*, resulting in the inhibition of mycelial growth and disease symptoms. In subsequent studies, various dsRNA constructs complementary to one or more *CYP51* genes also demonstrated varying degrees of resistance to *F. graminearum* infection in transgenic barley (Höfle et al., 2020; Koch et al., 2019).

Over the past decade, more target genes have been selected for HIGS to enhance plant resistance against *Fusarium* pathogens (reviewed in Attia et al., 2022; Machado et al., 2018; Ray et al., 2022). These genes were selected based on reported results of KO or KD mutants in various *Fusarium* species. The function of these genes includes the synthesis of hyphal walls, mycotoxin production, transcription regulation and protein kinase synthesis, and their KO or KD disrupts different stages of the infection cycle. In brief, in *F. graminearum Fg00677* encodes an alpha catalytic subunit of casein kinase, and *Fg08731* encodes a casein kinase 1; they were selected to enhance resistance of *B. distachyon* (He et al., 2019). In *F. graminearum*, three segments of *CHS3b* (CHS3b‐1, ‐3 and ‐5), a tandem DNA fragment FgSGE1‐STE12‐PP1, as well as *TRI5*, *GPMK1*, *FgCHY1*, *FgSR* and *FgTEAA* were used as target genes to enhance wheat resistance (Cheng et al., 2015; Shuai et al., 2024; Wang et al., 2020). However, Gao et al. (2024) recently reported no significant differences in disease severity and DON concentration between wild‐type and HIGS transgenic barley lines carrying a *TRI6* dsRNA construct under the used experimental conditions.

In *F. culmorum, FcGLS1* (*Glucan synthase 1*), *FcFMK1* (*Mitogen‐activated protein kinase*) and *FcCHSV* (*Chitin synthase V*) were identified to enhance wheat resistance (Chen et al., 2016). In *F. oxysporum, CYP51B* enhanced the resistance of soybean (Pérez et al., 2021). In *F. oxysporum* f. sp. *cubense, VEL*, *FTF1, EGR6* and *EGR11* target genes were selected to enhance banana resistance (Dou et al., 2020; Ghag et al., 2014). In *F. oxysporum* f. sp. *conglutinans, FOW2* (a putative transcription regulator), *FRP1* (*F‐box protein*) and *OPR* (*12‐oxo‐phytodienoate reductase homologous protein*) were used to enhance *Arabidopsis* resistance (Hu et al., 2015). In *F. oxysporum* f. sp. *lycopersici*, *FOW2*, *CHSV* (*Chitin synthase V*), *ODC* (*Ornithine decarboxylase*), *PEX6*, *GAS1* (*β‐1,3‐glucanosyltransferases*) and three fasciclin‐like protein genes *FoFLP1*, *FoFLP4* and *FoFLP5* enhanced tomato resistance (Bharti et al., 2017; Chauhan & Rajam, 2024; Singh et al., 2020; Tetorya & Rajam, 2021) (Table 2).

Even though these genes have been identified as effective targets for HIGS, the elucidation of additional target genes is desirable (Ray et al., 2022). Functions of many *Fusarium* genes have been validated (Niu et al., 2024; Rampersad, 2020; Xu et al., 2022) and the rapid development of sequencing technologies and gene function prediction tools in recent years has provided abundant resources for selecting more suitable target genes. However, despite substantial gene resources, some of them may face challenges such as restricted comparability of their functions among different pathogenic species or species‐specific functional overlap with gene paralogues. This scenario undoubtedly increases the difficulty of finding suitable target genes with broad‐spectrum antifungal activity. Furthermore, the same target genes may contribute differently to pathogenicity when in different host plants. For example, Fan et al. (2013) demonstrated that *FgCYP51C* is specifically required for full virulence on wheat ears, but not on *A. thaliana* floral tissue or fruits of apple and tomato, and targeting *FgCYP51C* by HIGS in *A. thaliana* and barley leaves did not reduce the fungal infection (Koch et al., 2019). By contrast, *FgCYP51B* is not required for full virulence on wheat ears (Fan et al., 2013), but targeting it by HIGS in *A. thaliana* and barley leaves significantly reduced the infection area (Koch et al., 2019). *FgDCL2* meets the same situation: its impact on pathogenicity varies significantly when infecting detached barley versus *B. distachyon* leaves (Koch et al., 2016; Werner et al., 2021). As shown by comparative transcriptomics, some fungal genes are equally expressed in *F. graminearum* when infecting maize, wheat or barley, whereas others are preferentially expressed only in the interaction of the fungus with one specific host (Harris et al., 2016). This certainly poses challenges to the utilization of model plants such as *A. thaliana* and *B. distachyon* for screening suitable RNAi target genes for other plants. Thus, it is highly recommended to conduct a large‐scope selection of RNAi target genes for a specific plant against a specific *Fusarium* species.

Overall, the HIGS strategy is highly effective, although it has some shortcomings, in particular the low acceptance of genetically modified organisms (GMOs) by the European public (Arpaia et al., 2020). From this perspective, it is understandable that SIGS has emerged as an attractive alternative (Koch et al., 2019).

### 3.5 Control of *Fusarium* by MIGS

In an open field, the primary challenge that dsRNAs/siRNAs face is short‐term environmental stability with long‐term pathogen infection (Hoang et al., 2022). Nanomaterials have been exploited to overcome this problem, but at the same time, they may bring unexpected impacts on complex microbial ecosystems (Hochella et al., 2019). Another challenge facing SIGS application is the delivery of RNA to the fungal infection sites, especially in the roots. As an alternative, Wen et al. (2023) developed a MIGS technology, which not only avoids using nanomaterials but also ensures the consistent production of dsRNAs/siRNAs by a rhizosphere soil microorganism.

In the MIGS system, siRNAs are produced in a donor fungus and then transferred to a recipient fungus to silence the target gene. The rhizospheric beneficial fungus *Trichoderma harzianum* was chosen as a donor, engineered to produce siRNAs from an inverted‐repeat RNAi construct. *F. oxysporum FoPMT2* (Table 2), which was selected as the target gene, encodes an O‐mannosyltransferase, and it has been proven to play essential roles in mycelial growth, conidiation, cell wall integrity and virulence (Xu et al., 2020). The RNAi constructs of three fragments corresponding to different regions of *FoPMT2* were introduced to *T. harzianum* for creating the engineered transformants Th‐Pmt2iFo, and these transformants were capable of producing siRNAs (Figure 1e). On solid medium, Th‐Pmt2iFo transformants suppressed the growth of *F. oxysporum*. When applied to rice, Th‐Pmt2iFo exhibited a strong protective effect against *F. oxysporum*. These results open up possibilities of MIGS to protect rice from the soilborne fungal pathogen *F. oxysporum*. Nonetheless, the key factors of interspecies RNAi between *T. harzianum* and *F. oxysporum* remain poorly understood (Fang, 2024; Wen et al., 2023).

### 3.6 Control of *Fusarium* infections by mycovirus‐based VIGS

Fusarium graminearum gemytripvirus 1 (FgGMTV1) is the first reported tripartite, circular single‐stranded DNA mycovirus (Li et al., 2020). A *F. graminearum* strain infected with FgGMTV1 produced a large number of viral siRNAs, suggesting that FgGMTV1 triggers RNA silencing (Wang et al., 2022). Recently, Zhang and colleagues developed a mycovirus‐based VIGS system for controlling FHB (Zhang, Wang, et al., 2023). In this study, *F. graminearum* was transfected with the FgGMTV1‐based VIGS vector p26‐D4, while it maintained normal fungal biology and wheat infectivity (Figure 1f). Based on the identified gene functions, eight genes associated with pathogenicity or DON biosynthesis have been chosen for insertion into the p26‐D4 vector, including *TRI1* (a cytochrome P450 oxygenase), *TRI5* (a trichodiene synthase), *TRI10* (a regulatory gene in trichothecene mycotoxin‐producing), *TRI101* (*C‐3 acetyltransferase*), *FgP1* (*WOR1‐like*), *FgPP1* and *FgCYP51C* (Table 2). Under laboratory conditions, strains carrying the p26‐D4 vector with an insert of 150 nt of these genes exhibited significantly lower virulence and DON production on wheat heads.

Under field conditions, two hypovirulent *F. graminearum* stains, generated by transforming the p26‐D4 vector with *TRI101* and *FgPP1* segments, have been utilized as biological control agents. When applying mycelium plugs of hypovirulent stains together with wild‐type conidial suspension to wheat spikes, the FHB disease symptoms and DON contamination were significantly decreased. In addition, pretreatment of wheat spikes with hyphal suspensions of these hypovirulent strains, followed by inoculation with the wild‐type 24 h later, also limited the FHB symptoms from locally treated spikelets to adjacent ones (Figure 1f). The reason the hypovirulent strains have this biocontrol ability is that mycovirus from the hypovirulent strain can be transmitted horizontally to virulent strains through hyphal anastomosis in the field (Zhang, Wang, et al., 2023).

This mycovirus‐based VIGS system provides a promising strategy for controlling FHB without relying on resistance genes. Before its commercialization, additional assessments must be conducted, such as the maintenance of high RNAi efficiency under field conditions, the duration of the silencing effects, the changes in agronomic properties of treated plants and the induction of the plant immune system (Zhang, Shi, et al., 2023).

## 4 CONCLUSION

A complete understanding of the RNAi components and mechanisms in *Fusarium* species is vital for advancing RNAi‐based disease control applications. While the mechanistic understanding of the *Fusarium* RNAi pathway is incomplete, to understand how *Fusarium* can use this pathway to regulate various biological processes may provide insights into the mechanisms involved.

In *Fusarium*, endogenous dsRNA can be used for identifying gene functions and selecting suitable target genes for further RNAi‐based control applications. Exogenous sRNAs can then be employed to test in vitro cultures and verify whether dsRNAs/siRNAs derived from selected target genes can inhibit fungal growth and whether they possess broad‐spectrum antifungal activity.

SIGS is a more time‐saving and GMO‐free method that can be applied more flexibly to emerging plant protection problems in a reasonable time frame, while HIGS is an environmentally friendly and highly efficient strategy to improve plant resistance by simultaneously silencing one or more fungal genes. MIGS protects plants from rhizospheric pathogenic microorganisms by employing donor transgenic microorganisms to continuously provide siRNAs; this strategy required detailed risk analysis and more basic research to identify its full potential. VIGS allows mycovirus‐based vectors containing segments of target genes to be horizontally transmitted from hypovirulent strains to virulent strains, producing dsRNAs/siRNAs to protect plants in the field. The RNAi‐based control strategies are currently evolving; they bring unprecedented and unlimited possibilities for future control of *Fusarium* diseases. Overall, however, there is not enough field data in the current literature to demonstrate the potential of RNAi strategies as a replacement for chemical pesticides under field conditions. Therefore, more field research is needed to tease out the full potential of RNAi‐based crop protection.

## Data Availability

Data sharing is not applicable to this article as no new data were created.
